# Blood-Brain Barrier Protein Claudin-5 Expressed in Paired *Xenopus laevis* Oocytes Mediates Cell-Cell Interaction

**DOI:** 10.3389/fphys.2020.00857

**Published:** 2020-07-21

**Authors:** Nora Brunner, Laura Stein, Valeria Cornelius, Ria Knittel, Petra Fallier-Becker, Salah Amasheh

**Affiliations:** ^1^Department of Veterinary Medicine, Institute of Veterinary Physiology, Freie Universität Berlin, Berlin, Germany; ^2^Institute of Pathology and Neuropathology, University Hospital of Tuebingen, Eberhard Karls University of Tuebingen, Tuebingen, Germany

**Keywords:** tight junction, claudins, blood-brain-barrier, sodium caprate, *Xenopus laevis* oocyte

## Abstract

Claudin-5 determines the sealing properties of blood-brain barrier tight junctions and its function is impaired in neurodegenerative and neuroinflammatory disorders. Focusing on the contribution of claudin-5 to the *trans*-interaction within the tight junction seal, we used *Xenopus laevis* oocytes as an expression system. Cells were clustered and challenged in a novel approach for the analysis of claudin interaction. We evaluated the strengthening effect of claudin-5 to cell-cell-connection in comparison to claudin-3. Application of a hydrostatic pressure impulse on clustered control oocyte pairs revealed a reduction of contact areas. In contrast, combinations with both oocytes expressing claudins maintained an enhanced connection between the cells (cldn5–cldn5, cldn3–cldn3). Strength of interaction was increased by both claudin-3 and claudin-5. This novel approach allowed an analysis of single claudins contributing to tight junction integrity, characterizing homophilic and hetrophilic *trans*-interaction of claudins. To test a new screening approach for barrier effectors, exemplarily, this 2-cell model of oocytes was used to analyze the effect of the absorption enhancer sodium caprate on the oocyte pairs.

## Introduction

The tight junction protein family is crucial for cell physiology as lack or impairment is associated with diseases and dysfunction of many organs and tissues, as shown e.g., in the inner ear (Wilcox et al., 2001; Florian et al., 2003), kidney (Konrad et al., 2006; Günzel et al., 2009), gastrointestinal tract (Resnick et al., 2005; Amasheh et al., 2009), epidermis (Furuse et al., 2002; Tebbe et al., 2002), and brain capillaries (Nitta et al., 2003; Wolburg et al., 2003). Claudins represent a transmembrane protein family comprising at least 27 members (Mineta et al., 2011). In addition to their four transmembrane helix domains, they contain two extracellular loops (ECL1 and ECL2), a short N-terminus and a C-terminus (Suzuki et al., 2014). Specific claudin expression patterns determine and reflect the selective permeability of epithelia, and the ability of claudin proteins to interact in *cis* (within the same membrane) and in *trans* (between the membranes of the neighboring cell) allows the formation of barrier forming and pore forming tight junction strands (Van Itallie and Anderson, 2006).

Claudin-5 is strongly expressed in capillary endothelia and dominates the tight junction (TJ) of the blood-brain barrier (BBB) as the expression is >100 times higher compared to any other claudin (Ohtsuki et al., 2007). Moreover, it is expressed in a variety of epithelial tissues including lung (Soini, 2011), exocrine tissues (Comper et al., 2009), intestinal (Garcia-Hernandez et al., 2017), and urinary tract (Koda et al., 2011). However, claudin-5 causes a stronger barrier in brain capillaries than in other tissues (Reinhold and Rittner, 2017) and its function is impaired in neurodegenerative and neuroinflammatory disorders (Greene et al., 2019). Hence, claudin-5 is crucial for maintaining the BBB. But the BBB is not only protective, it also limits the therapeutic options as drugs are hindered to permeate this barrier.

Nitta et al. (2003) reported, that the BBB is more permeable to molecules of 800 Da in size in claudin-5 deficient mice compared to wild type mice (Nitta et al., 2003). This was in accordance with transfection experiments demonstrating a sealing effect of claudin-5 in Caco-2 cell monolayers (Amasheh et al., 2005).

Another major barrier-forming claudin is claudin-3, which has been reported to selectively seal the barrier against the passage of ions of either charge and uncharged solutes (Milatz et al., 2010). It is also expressed in the endothelial tight junction of brain capillaries and its functional loss is observed in phases of microvessel inflammation, glioblastoma and choroid plexus of patients with multiple sclerosis (Engelhardt et al., 2001; Wolburg et al., 2003).

Barrier properties can be dynamically modified, as e.g., incubation with sodium caprate was demonstrated to rapidly and reversibly decrease transepithelial electrical resistance in the human intestinal cell line HT-29/B6 (Krug et al., 2013). Sodium caprate transiently opens claudin-5 containing barriers at tight junctions of epithelial and endothelial cells (Del Vecchio et al., 2012).

This indicates, that claudin-5 is a promising target for drug delivery enhancement in the BBB.

In this study, we aimed to employ the heterologous expression system of *Xenopus laevis* oocytes (Vitzthum et al., 2019) for the analysis of claudin-5 and claudin-3 interaction and pertubation. Due to the lack of endogenous cell-cell-contacts, this single cell expression system enables the analysis of specific claudins without interference of other tight junction proteins.

## Materials and Methods

### Harvest of Oocytes and cRNA Microinjection

Oocytes were collected from adult female African claw frogs by surgical laparotomy. For anasthesia, 0.2% MS222 (ethyl 3-aminobenzoate methanesulfonate, Sigma-Aldrich, Taufkirchen, Germany) was used as a bath solution for 5–10 min at 20°C. Once surgical anasthesia was reached, skin and abdominal muscle incisions were made and ovarian mass was exteriorized and ovarial tissue removed. The isolation of oocytes was conducted by enzymatic digestion at room temperature for 90 min in 1.5 mg/ml collagenase Fisher BioReagents BP2649-1 (Fisher Scientific, Schwerte, Germany) dissolved in oocyte Ringer solution (ORi) as described by Vitzthum et al. (2019). Follicular cells were removed by incubation in Ca^2+^-free ORi containing (in mM): NaCl (90), KCl (1), EGTA (triethylene glycol diamine tetraacetic acid) (1), 5 HEPES (5); pH 7.4 for 10 min on a mechanical shaker with 50 rpm. Oocytes of stages V and VI (>1000 μm) were injected (Nanoliter 2010, World Precision Instruments, Sarasota, FL, United States) with 1 ng cRNA encoding human claudin-5, claudin-3 or RNase-free water as controls. Injection volume was 50.6 nl per oocyte. After injection, oocytes were incubated at 16°C in ORi 3 days for protein expression.

### Isolation of Membrane Fractions and Immunoblotting

Ten injected oocytes were pooled for western blot analysis and resuspended in 500 μl homogenization buffer containing (in mM) MgCl_2_ (5), NaH_2_PO_4_ (5), EDTA (ethylenediaminetetraacetic acid) (1), sucrose (80), and Tris (Tris(hydroxymethyl)aminomethane) (20); pH 7.4. Oocyte extracts were centrifuged twice at 200 rpm for 10 min at 4°C to discard cell debris. The supernatant was centrifuged at 13,000 rpm for 30 min at 4°C to pellet the cell membrane as described by Leduc-Nadeau et al. (2007). Pellets were resuspended in 80 μl homogenization buffer. Protein quantification was done colorimetrically using Pierce 600 nm Protein Assay Kit (Thermo Fisher Scientific, Hennigsdorf, Germany) according to the manufacturer instruction in a 96 well plate. The plate reader (PerkinElmer EnSpire Multimode Plate Reader, Waltham, MA, United States) was adjusted to 562 nm and Bovine Serum Albumin Standard (Thermo Fisher Scientific, Hennigsdorf, Germany) ranging from 125 to 2000 μg/ml was employed for evaluation. Prior to immunoblotting, samples were mixed with 4× Laemmli buffer (Bio-Rad Laboratories, Munich, Germany), loaded onto a 10% SDS polyacrylamide gel and electrophoresed. For protein transfer, PVDF membranes were used and blocked in 5% non-fat dry milk in Tris-buffered saline for 120 min. Proteins were detected by immunoblotting using primary antibodies raised against claudin-3 or claudin-5 (invitrogen #35-2500, #34-1700, #34-1600, Life Technologies, Carlsbad, CA, United States).

Peroxidase-conjugated goat anti-rabbit and anti-mouse antibodies (#7074, #7076 Cell Signaling Technology, Danvers, MA, United States) were used to bind to the primary antibodies and therefore incubated for a minimum of 45 min at room temperature. For detection, Clarity Western ECL Blotting Substrate (#1705061, Bio-Rad Laboratories GmbH, Munich, Germany) was used and signals were visualized by a ChemiDoc MP system (Bio-Rad Laboratories).

### Immunohistochemistry

Injected oocytes were fixed in 4% PFA (16% paraformaldehyde, E15700, Science Service, Munich, Germany) for 4 h at room temperature followed by dehydration gradient from 70% ethanol to xylol (Carl Roth, Karlsruhe, Germany) within 48 h. Samples were embedded in paraffin and cross-sectioned (5 μm) by using a Leica RM 2245 microtome (Leica Microsystems Heidelberg, Germany). Shortly before immunohistochemical treatment, paraffin was removed via xylol to ethanol gradient. Non-specific binding sites were blocked using 5% goat serum in phosphate-buffered saline and incubated with the same primary antibodies as for immunoblotting. Samples were incubated with the secondary antibodies Alexa Fluor-488 goat anti-rabbit and Alexa Fluor-594 goat anti-mouse (Life Technologies, Carlsbad, CA, United States) and examined by confocal laser-scanning immunofluorescence microscopy (LSM 710, Zeiss, Oberkochen, Germany).

### Freeze Fracture Electron Microscopy

Freeze fracture electron microscopy was performed as reported recently (Greene et al., 2019). For fixation, injected oocytes were incubated in glutaraldehyde (2.5% in 0.1 M cacodylate buffer) overnight at 4°C. After washing with cacodylate buffer, oocytes were prepared for freeze fracturing. Samples were cryoprotected in 30% glycerol and frozen in liquid nitrogen. After fracturing, and shadowing with platinum and carbon (BAF400D; Balzers, Liechtenstein), remaining organic material was removed by a sodium hypochlorite wash. Oocytes were analyzed in a transmission electron microscope (EM-10, Zeiss, Oberkochen, Germany) and photographed with a digital camera (Tröndle GmbH). Morphometrical analysis of the tight junction strands was performed at a magnification of 20,000×.

### Paired-Oocyte Assay and Quantification of Contact Areas

Mannitol was implemented to shrink the injected oocytes and allow a mechanical removal of the vitelline membrane using forceps without damaging the plasma membrane. 5–10 oocytes were placed in a petri dish (35 mm diameter, Thermo Fisher, Henningsdorf, Germany, #153066) filled with ORi. Mannitol was added and dissolved until hypertonic shrinking of the cells was achieved (approximately 400 mOsmol/l for 10 min). After manual devitellinisation, oocytes were immediately transferred to a 24 well plate (1. 86 cm^2^ surface area, TPP Techno Plastic Products, Trasadingen, Switzerland, # 92024) containing 2 ml of ORi. In each well, two cells were gently clustered by pushing them together with a Pasteur pipette (1 ml, Thermo Fisher, Henningsdorf, Germany, #PP88SB) and a bulbous probe.

Oocyte pairs of claudin-5-expressing (cldn5 − cldn5), claudin-3-expressing (cldn3 − cldn3), claudin-3 and claudin-5 coexpressing (cldn3,5 − cldn3,5) and control oocytes (control − control) were kept together for up to 48 h in ORi at 16°C.

Bright field microscopy was employed for quantification of contact area of clustered oocytes after 1, 24, and 48 h. Images of the naïve oocyte pairs in 24 well culture dishes were taken at these time points using a Leica DMI6000 B Microscope (Leica Microsystems, Wetzlar, Germany). Diameter of contact area was measured using the micron scale (LAS-AF 3.2.0). Contact areas are regarded to be circular and thus the contact area was calculated by using the circle equation *A*=π∙*r*^2^.

### Hydrostatic Pressure Impulse Assay

Vitelline membranes were mechanically removed as described before and oocytes clustered analagous to the paired oocyte assay. Additionally, mixed oocyte pairs (control–cldn5 and control–cldn3) were tested in the hydrostatic pressure impulse (HPI) assay. After 24 h of stabilization, a defined hydrostatic impulse was created using a single channel electronic pipette (EE-300R, Eppendorf Research Pro, software version 2.06.00, Hamburg, Germany).

**FIGURE 1:**
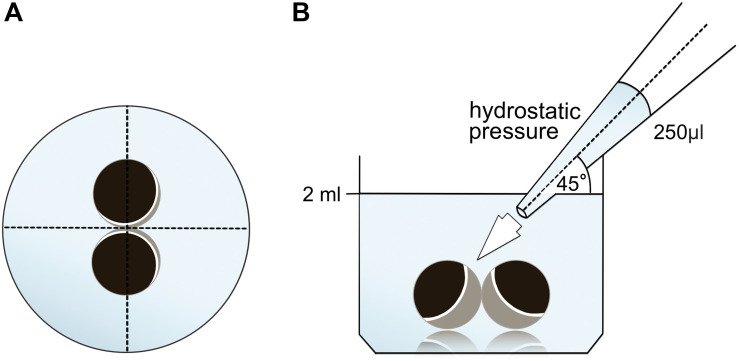
Experimental setup of hydrostatic pressure impulse assay **(A)** schematic top view of the well: central positioning of the oocyte pair was checked before application of the hydrostatic pressure. **(B)** Schematic side view of the well: 250 μl ORi was added using a single channel electronic pipette. The dispensing speed was uniformely set to maximum speed. The angle (45°) and distance of application was uniformely applied. Ambient pressure, viscosity of ORi and diameter of pipette tip opening were kept under constant conditions.

Oocytes were kept in 24 well plates containing 2 ml ORi and central positioning was checked before application of the pipetting volume 250 μl ORi. The dispensing speed was uniformely set to maximum speed, equating a dispensing speed of 0.9 s. Furthermore, the angle (45°) and distance of application (∼1.3 cm) was uniformely applied. Ambient pressure, viscosity of the ORi and diameter of pipette tip opening were kept under constant conditions. Bright field microscopy was employed for quantification of contact areas 30 min after the hydrostatic pressure was applied and compared to contact areas before application. The experimental setup is described in Figure 1.

### Caprate Incubation

For caprate incubation, sodium caprate (#C4151, Sigma Aldrich, Taufkirchen, Germany) in final concentrations of 50, 100, and 500 μM, or ORi as reference group, was added to the oocytes 24 h after pairing. Oocytes were kept in 24 well plates containing 2 ml ORi and caprate solution was dissolved in a defined addition volume of 250 μl ORi per well. Width of contact area was quantified at 30, 60, and 120 min after addition.

### Statistical Analysis

Statistical analysis was performed with JMP Pro 14.0.0 (NC, United States). Data are presented as medians and displayed as percentual change based on the clustered combination at the first examination points. Figures 4, 5 are presented as Box plots, depicting the first quartile (25-percent), the median (50-percent) and the second quartile (75-percent). The whiskers are drawn down to the 10th percentile and up to the 90th percentile. Normal distribution was checked by using Shapiro–Wilk-test.

Kruskal–Wallis test was used for multiple comparison, followed by a Dunn-Bonferroni correction. *p*-values are given as continous numbers.

## Results

### Expression of Claudin-5 and Integration Into *X. laevis* Oocyte Plasma Membrane

**FIGURE 2:**
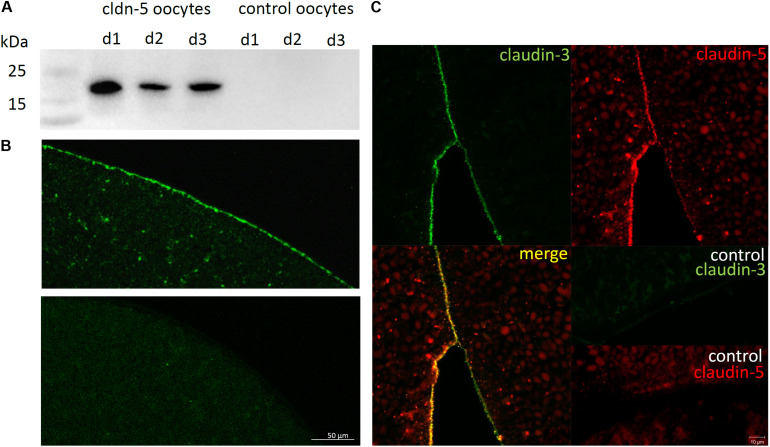
Detection of heterologously expressed claudins in *Xenopus laevis* oocytes. **(A)** Immunoblot analysis of tight junction protein claudin-5 in *X. laevis* oocytes of three animals (d1–d3). Cell membrane lysates were subjected to SDS-PAGE followed by immunoblot onto PVDF membranes. The membranes were incubated with primary antibodies and secondary peroxidase-conjugated antibodies (*n* = 3). **(B)** Immunofluorescent staining revealed specific claudin-5 signals (green) in oocyte membranes of all cRNA-injected oocytes, whereas in water-injected controls, no claudin-specific signals were detected in confocal microscopy. Representative images of oocytes derived from three animals. Scale bars: 50 μm. **(C)** Immunofluorescent staining of claudin-5 and claudin-3 expressing oocytes revealed specific claudin-3 signals (green) und claudin-5 signals (red) in oocyte membranes of cRNA-injected oocytes, whereas in water-injected controls, no claudin-specific signals were detected. Colocalization of expressed claudin proteins within the oocyte plasma membrane is revealed by double immunofluorescent staining (yellow). Scale bar: 10 μm.

To test the successful expression and integration of the tight junction protein claudin-5 into the oocyte plasma membrane, 3 days after injection of claudin-5 cRNA, membrane fractions were analyzed by immunoblotting. All samples from three individual animals (d1–d3) revealed claudin-5 specific signals at 23 kDa, whereas RNAse-free water-injected oocytes showed no specific signal for claudin-5 expression (Figure 2A).

For visualization of the expressed proteins within the plasma membrane, immunohistochemical stainings were performed and analyzed by confocal laser scanning microscopy (Figure 2B). Specific signals were detected and evenly distributed throughout the plasma membrane of claudin-5 expressing oocytes. In accordance with immunoblots, no specific signals were detected in control oocyte plasma membranes.

Thus, after injection of cRNA, claudin-5 was successfully expressed and integrated in the plasma membrane of *X. laevis* oocytes.

Co-expression of claudin-3 and claudin-5 in oocyte pairs revealed specific signals for claudin-5 (red) and claudin-3 (green) in both cells (Figure 2C). Oocyte plasma membranes showed a fusion of the neighboring cells provided by direct cldn3,5–cldn3,5 interaction (yellow).

### Patches of Tight-Junction Strands Are Visible in Claudin-5 Expressing Oocytes

**FIGURE 3:**
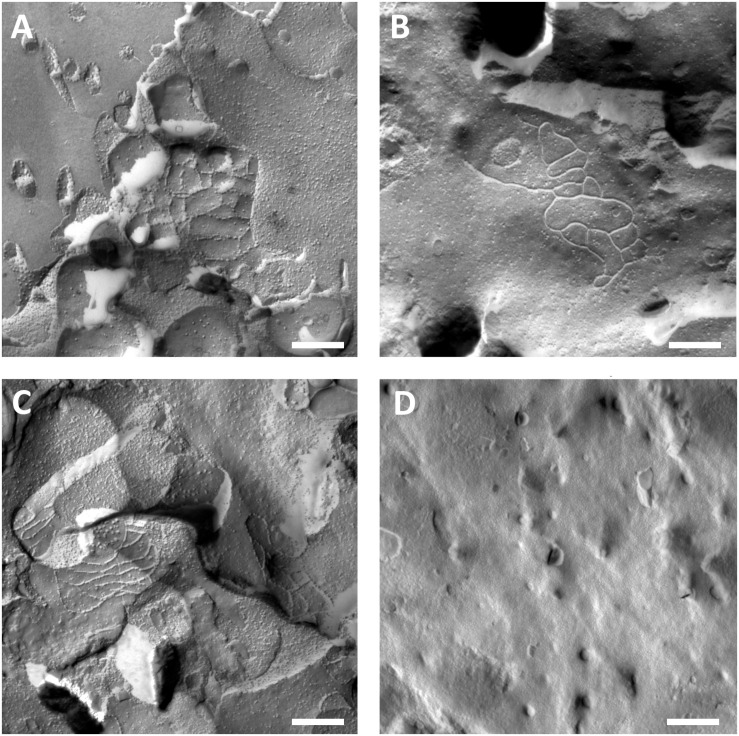
Freeze fracture electron microscopy. **(A)** Freeze fracture electron microscopy reveals tight junction protein cldn-5 as a meshwork of angular discontinous fibrils in rows in *Xenopus laevis* oocytes. **(B)** Freeze fracture electron microscopy reveals tight junction protein cldn-3 as a meshwork of rounded fibrils in *X. laevis* oocytes. **(C)** Freeze fracture electron microscopy of claudin-3 and claudin-5 coexpressing oocytes reveal fibrils that both bear properties of claudin-3 and claudin-5. **(D)** Water injected control oocytes have a smooth surface. Representative images of oocytes derived from three animals. Scale bar: 250 nm.

Oocyte plasma membranes were analyzed and visualization of tight-junction strands was successful (Figure 3). Freeze fracture electron microscopy showed patches of strand morphology in the plasma membranes of claudin-5 injected oocytes, and strand organization of claudin-5 expressing oocytes was highly organized and of angular shape (Figure 3A). Tight junction strands were primarily detected in the in the protoplasmic (P-) face of the membrane. Claudin-3 injected oocytes showed rounded highly organized tight junction strands as reported previously (Vitzthum et al., 2019; Figure 3B). Freeze fracture electron microscopy of claudin-3 and claudin-5 coexpressing oocytes revealed fibrils that both bear properties of claudin-3 and claudin-5. Fibril strand architecture of coexpressing oocytes appeared both rounded and complex as claudin-3 expressing oocytes, but also discontinous and more angled as shown for claudin-5 expressing cells (Figure 3C). Control oocytes had a typical smooth surface (Figure 3D).

**FIGURE 4:**
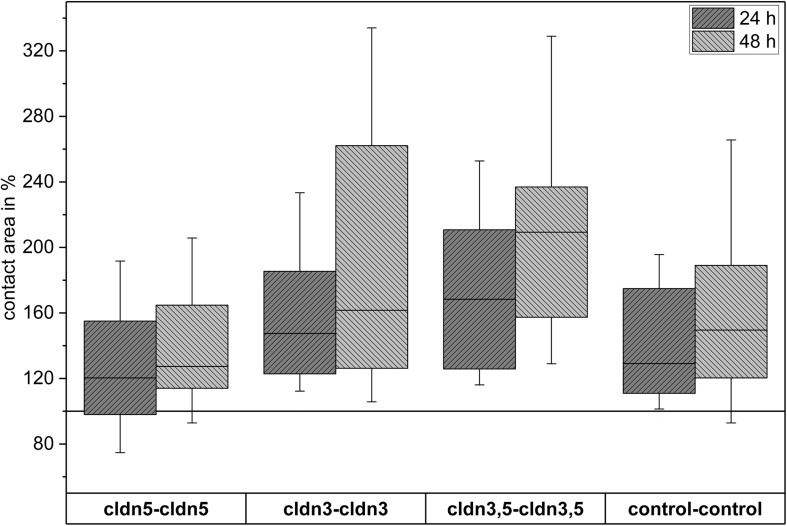
Median contact areas of clustered oocyte combinations cldn5–cldn5, cldn3–cldn3, cldn3,5–cldn3,5 and control–control 24 and 48 h after clustering in % of initial contact areas shortly after clustering (*n* = 8–38).

**FIGURE 5:**
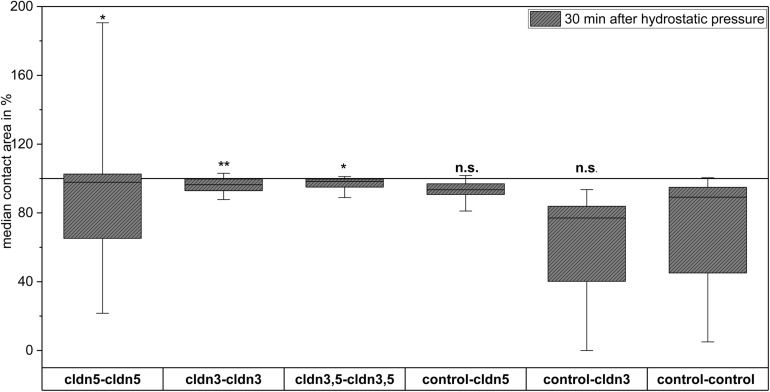
Contact areas of claudin-5, claudin-3, and coexpressing claudin-3 and claudin-5 oocytes in hydrostatic pressure impulse (HPI) challenge after stabilization period and 30 min after HPI (*n* = 16–70, *p < 0.05, **p < 0.01, Kruskal-Wallis followed by a Dunn–Bonferroni correction).

### Paired Oocyte Assay for Analysis of Claudin *Trans*-Interaction

All clustered combinations showed a time-dependent increase in contact area over the measured period of time (Figure 4 and Table 1).

**TABLE 1:** Oocyte contact areas within 48 h after clustering.

Clustered combination	Time point	Median contact area in %	*n*
cldn5-cldn5	1 h	100	33
	24 h	120	33
	48 h	127	33
cldn3-cldn3	1 h	100	29
	24 h	147	29
	48 h	162	29
cldn3,5-cldn3,5	1 h	100	8
	24 h	168	8
	48 h	209	8
control-control	1 h	100	38
	24 h	129	38
	48 h	150	38

The contact area of water-injected control oocytes increased to 129% after 24 h and 150% after 48 h. Clustered pairs of oocytes expressing claudin-3 also showed an increase of contact areas to 147% (24 h) and 162% (48 h). Clustered pairs of oocytes coexpressing claudin-3 and claudin-5 showed contact areas of 168% (24 h) and 209% (48 h). Clustered pairs of oocytes expressing claudin-5 alone showed contact areas of 120% (24 h) and 127% (48 h). Therefore contact areas in all tested combinations were comparable.

### Hydrostatic Pressure Impulse Assay Reveals Claudin-Specific Junction of Oocyte Pairs

In a separate approach, oocytes expressing claudin-3 or claudin-5 or coexpressing both claudins were clustered after mechanical devitellinization. Oocytes were challenged by employing a HPI and contact areas were measured and calculated 30 min after challenge and compared to initial areas after the 24 h stabilization period (Figure 5 and Table 2). After hydrostatic pressure challenge, the contact area of water-injected control oocytes decreased to 89%. Clustered pairs of oocytes expressing claudin-5, claudin-3 or coexpressing claudin-3 and claudin-5 retained larger contact areas (97%, *p* = 0.0235; 96%, *p* = 0.003; 98%, *p* = 0.0253). The contact areas of mixed water-injected control oocytes and claudin-expressing oocytes (control–cldn5 and control–cldn3) did not significantly differ from control oocytes (93%, *p* = 0.2900; 83%, *p* = 0.4455).

**TABLE 2:** Hydrostatic pressure impulse assay.

Clustered combination	Median contact area in %	*n*
cldn5-cldn5	97	45
cldn3-cldn3	96	44
cldn3,5-cldn3,5	98	16
control-cldn5	93	17
control-cldn3	83	19
control-control	89	70

### Incubation With Caprate

In a pilot incubation experiment, oocytes were injected and paired in combinations either expressing claudin-5 (cldn5–cldn5) or injected with RNAse free water as controls (control–control). Pairs were incubated with final sodium caprate concentrations of 50, 100, or 500 μM. The incubation of oocyte pairs with ORi served as a reference group. Oocytes were clustered and after 24 h of stabilization, incubation started and contact widths were measured 30, 60, and 120 min after addition (Supplementary Figure S1).

The addition of ORi resulted in an initial decrease of contact areas both in claudin-expressing and water-injected oocyte pairs that is dispersed 60 or 120 min after addition. This is outlined by the parabolic shape of the connection line between the median contact areas over time (red curves in Supplementary Figure S1). However, incubation with 100 and 500 μM sodium caprate increased contact areas slightly (100 μM) or strongly (500 μM) 30 min after caprate addition from 5.1 × 10^5^ to 5.2 × 10^5^ μm^2^ and 4.3 × 10^5^ to 4.9 × 10^5^ μm^2^.

## Discussion

In the present study, we employed the classic model for transporters and human disease modeling (Tammaro et al., 2009; Nenni et al., 2019), the *X. laevis* oocytes, for an in-depth analysis of claudin-5 interaction and functional contribution to the junction seal. To this end, a novel approach, introducing a HPI for challenging interaction within the contact area of clustered *Xenopus* oocytes, was established.

### Claudins Contribute to Stronger Adhesion Properties

In accordance with previous results from Vitzthum et al. (2019), single claudins expressed in oocytes did not lead to an increase of interaction contact areas compared to control oocytes. However, immunoblot and immunohistochemical visualization proved the successful expression and integration into *X. laevis* oocyte plasma membrane. Furthermore, the use of confocal laser scanning microscopy allowed a precise localization of the expressed claudins in the plasma membrane as the pinhole blocked out-of-focus fluorescence. A quantification of immunohistochemical signals was not pursued, as the affinity of antibodies for binding their targets differs.

Vedula et al. (2009) used a micropipette aspiration technique to investigate aspects of claudin-claudin interaction using L-fibroblasts transfected with GFP-tagged occludin, cldn-1, and cldn-2. The separation force needed to detach two cells from each other was larger in cldn-1 and cldn-2 transfected cells (∼2.8 and 2.3 nN, respectively). Though this approach might appear also promising for the claudin-claudin- interaction analysis of expressing oocytes, preliminary tests revealed that a detachment of clustered oocytes is not possible without disruption of the oocyte plasma membranes.

Therefore, as a novel approach, the force of the connections was measured by a HPI. Although the HPI does not provide a quantification of the separation force in absolute values (e.g., in newton’s), it allowes a quick and cost-effective analysis of the claudin interaction without disturbance of other tight junction proteins (e.g., occludin, tricellulin, JAM-A). Claudins contribute to the junction of oocyte pairs as they show a larger contact area compared to water-injected oocytes after HPI. This indicates strong homophilic *trans*-interaction between the claudin-expressing cells.

Strand fibril architecture is specific for single claudins (Colegio et al., 2002). In freeze fracture electron microscopy, claudin-3 was reported to assemble a more rounded strand meshwork in loop shapes in *Xenopus* oocytes (Vitzthum et al., 2019; Figure 3B) while in our study, claudin-5 formed a meshwork more angular and ordered in rows. The images revealed, that the tight junction protein claudin-5 forms a meshwork of fibrils in discontinous, angular shaped rows in *X. laevis* oocytes. This is in accordance with claudin-5 strands known to occur as chains of particles associated to the P-Phase (Piontek et al., 2011). In our experiments, geometrical shape of the fibrils seemed to have no effect on the oocytes adhesion properties. In accordance with that, paracellular resistance was reported to be unrelated to fibril number and fibril-forming properties (Colegio et al., 2002). Furthermore, claudin-3 and claudin-5 are shown to have a similar capability for homophilic *trans*-interaction in HEK293 cells (Piontek et al., 2011).

This model of *X. laevis* oocytes, expressing single tight junction proteins, allows an observation of the effect of substances like sodium caprate on the formation of contact areas between clustered oocytes. It may therefore provide a useful tool for a time and cost-efficient screening for substances affecting the tight junction barrier.

Sodium caprate concentrations of 100 and 500 μM may convey a protective effect on claudin-5 expressing oocytes, resulting in increasing contact areas after 30 min of incubation.

Krug et al. (2013) demonstrated, that incubation with sodium caprate led to a rapid and reversible decrease of transepithelial resistance in human intestinal cell line HT-29/B6. Furthermore, confocal laser-scanning microscopy revealed a marked reduction of claudin-5 in HT-29/B6 cells treated with the medium chain fatty acid laurate (Dittmann et al., 2014). The first extracellular loop of claudins (ECL1) is important for the barrier properties of the tight junction, while the second extracellular loop (ECL2) is involved in strand formation of *trans*-interaction (Piontek et al., 2008; Rossa et al., 2014; Greene et al., 2019). Claudin-5 binders targeting ECL1 or ECL2 of claudin-5 may induce intracellular uptake of the tight junction protein, thereby thwarting the claudin-5 *trans*-interactions in the tight junction seal between adjacent cells and loosening the paracellular space (Hashimoto et al., 2017).

Thus, we hypothesized that incubation with sodium caprate would led to a decrease in contact area of clustered claudin-5 expressing *X. laevis* oocytes. Unexpectedly, increasing concentrations of sodium caprate (100 and 500 μM) led to increasing contact areas of clustered oocytes expressing claudin-5 indicating a protective effect of sodium caprate on the tight junction seal. Furthermore, sodium caprate is described to induce contraction of the actomyosin perijunctional ring, widening the paracellular space (Lindmark et al., 1998; Maher et al., 2009). This effect is based on the phosphorylation of the regulatory light chain of myosin via a phospholipase C activation which leads to a cleavage of phosphatidylinositol 4,5-bisphosphate (PIP2) into inositol triphosphate (IP3) and diacylglycerol (Tomita et al., 1995). The tight junction complex linked by the scaffolding protein ZO-1 to the actin cytoskeleton is then redistributed from the tight junction to the cytoplasm (Lindmark et al., 1998; Turner, 2000). The cytoarchitecture of the *X. laevis* oocyte is crucial for cytoplasmic regionalization during oogenesis (Wylie et al., 1985). Though, prior to fertilization, tjp-1 gene expression, the gene encoding for ZO-1 is only expressed 1.9 TPM in oocyte stages V–VI (Session et al., 2016). A reduced interaction of the claudins with the cytoskeletal scaffold may therefore explain the unexpected result of the caprate incubation. Is the scope of future studies to verify the mechanistic basis of the finding.

However, a variant effect of sodium caprate on the paracellular permeability was described in literature before. In Peyer’s Patch tissue taken from the intestine of adult pigs, a similar strengthening effect was detected. Claudin-5 was significantly increased after incubation with 5 mM caprate. In this study caprate led to a significantly higher transepithelial electrical resistance (TEER) in the follicle associated epithelium (Radloff et al., 2019).

## Conclusion

In conclusion, heterologous expression of the tight junction protein claudin-5 in *X. laevis* oocytes allows new insights into the contribution of single claudins to cell-cell interaction and adhesions properties of adjacent cells. Thus, use of the *X. laevis* tight junction model for claudin-5 allows analysis of BBB components in a single-cell model.

## Data Availability Statement

The datasets generated for this study are available on request to the corresponding author.

## Ethics Statement

The animal treatments were in accordance with the guidelines of German legislation, with approval by the animal welfare officer for the Freie Universität Berlin and under the governance of the Berlin Veterinary Health Inspectorate (Landesamt für Gesundheit und Soziales Berlin, permit G0025/16).

## Author Contributions

All authors have read and approved the manuscript. NB and SA designed, planned, and supervised the experiments and wrote the manuscript. NB, LS, VC, RK, and PF-B performed the experiments and data analysis.

## Conflict of Interest

The authors declare that the research was conducted in the absence of any commercial or financial relationships that could be construed as a potential conflict of interest.

## Supplementary Material

FIGURE S1 Contact areas of claudin-5 expressing oocyte pairs in μm^2^ during incubation with sodium caprate in different concentrations (*n* = 6–8, respectively) and water-injected oocytes as controls (*n* = 5–7, respectively).
